# By the Light of Day: Quality, Safety, and Education During the Overnight Admission Handoff

**DOI:** 10.7759/cureus.4529

**Published:** 2019-04-23

**Authors:** Ganesh P Devendra, Gabriel M Ortiz, Lawrence A Haber

**Affiliations:** 1 Hospital Medicine, Oregon Health and Sciences University, Portland, USA; 2 Internal Medicine, University of California San Francisco, San Francisco, USA; 3 Internal Medicine - Hospital Based Medicine, University of California San Francisco, San Francisco, USA

**Keywords:** hospital medicine, nocturnists, resident education

## Abstract

Background

Current duty hour restrictions have led to increased patient handoffs as well as increased use of faculty in the nocturnist role. Nocturnists typically supervise residents and perform direct patient care leading to a diversity of provider experience level during morning handoffs. In this study, we explored how the presence of nocturnists impacts perceptions patient safety, quality, and educational value of morning care transitions.

Methods

We performed a cross-sectional survey examining the housestaff and attending perceptions of the morning sign-out of overnight admissions from both night float residents and nocturnists in July of 2016. Survey responses were Likert-style format, querying respondents’ level of agreement (1-5, strongly disagree to strongly agree) with statements. 108 providers responded (41% response rate)

Results

Relative to attendings, residents reported feeling like they had less time to ask questions (4.0 vs. 5.0, *p *< 0.001) and felt less comfortable asking questions of the nocturnist during handoff (4.0 vs. 5.0, *p *< 0.001). Residents were also less comfortable than attendings in changing a nocturnist’s plan of care (4.0 vs. 5.0, *p *< 0.001). Housestaff reported that receiving signout from the overnight resident was more likely to improve their confidence managing similar conditions (4.0 vs. 3.0, *p *< 0.001).

Conclusion

The benefits of nocturnist supervision may come at an educational cost as trainees feel less comfortable asking questions or changing the plan of care. With increasingly prevalent night float systems and nocturnist providers, academic programs have to negotiate the balancing safe and high-quality patient care with creating positive learning environments and clear expectations.

## Introduction

Verbal provider-to-provider handoffs are the most common mechanism for transitioning care in the inpatient setting [1]. Handoffs are used to report overnight events on previously admitted patients, and introduce providers to new admissions. The 2011 duty hour restrictions reduced inpatient resident shift length, leading to increased transitions of care between providers [2-3]. Increasingly frequent patient handoffs may jeopardize patient safety, as the process is variable, unstructured, and error prone [1,4-10]. Subsequently, there has been growing housestaff education focusing on improving and standardizing inpatient provider transitions [11-12].

Increased housestaff supervision mandates and duty hour restrictions have led academic training institutions to employ overnight in-hospital faculty (nocturnists) [13-15]. Nocturnists can provide direct overnight supervision of trainees as well as perform direct patient care duties to offload resident workload. Prior investigation has shown that residents found nocturnist presence enhanced the clinical value of the night float rotation, without compromising housestaff decision-making autonomy [16-18]. No prior studies have examined the morning transition of care of newly hospitalized patients in this current academic environment, where patient care by overnight resident and faculty physicians is transferred to daytime clinicians.

We examined perspectives of faculty and trainee daytime providers receiving new admissions from overnight hospitalists and residents with respect to the quality of information transmission, handoff safety, and educational experience of receiving physicians. Our goal was to identify barriers to safe, high-quality care of the newly hospitalized patient at academic institutions and inform best practices for standardizing this increasingly frequent transition of care.

## Materials and methods

Our study was conducted at a 183-bed urban safety-net medical center affiliated with an internal medicine residency program of 170 housestaff. During the resident “night float” rotation at our hospital, second- and third-year residents complete seven days of overnight shifts. Shift duration is 13 hours, beginning at 8 pm and ending at 9 am, and involves admitting general medicine patients to the floor and step-down units, as well as supervising the cross-covering medicine intern. Since 2011, direct overnight supervision of the nighttime housestaff has been provided by an on-site attending physician. This nocturnist also admits patients in a 1:1 ratio with the resident, manages transfer requests from outside hospitals, performs medicine consults, and organizes patient distribution to the day teams based on team census levels. The majority of these shifts are staffed by dedicated nocturnists, all junior faculty within the Division of Hospital Medicine, with approximately one-fifth of shifts staffed by moonlighting physicians who have completed residency training. Overnight admissions by both housestaff and nocturnist are then handed off to the day teams via a full presentation to residents, interns, and attending of the accepting team. Attending physicians from divisions of hospital medicine, general internal medicine, and subspecialists all staff our general medicine services. Time allotted to overnight presentations is one hour between 8 and 9 am with presentations typically taking place in a conference room, not at the bedside.

We administered a cross-sectional survey in July 2016 to all current internal medicine housestaff who cared for patients during day time on the inpatient general medicine service at our institution in the past four academic years (since the advent of our current the overnight system). We administered the same survey to internal medicine faculty who had attended on the general medicine teaching services in the past four years. Residents and faculty who had not worked at the hospital or who had not received overnight handoffs during the allotted time were excluded from the survey.

Surveys examined housestaff and attending perceptions of the morning sign-out of overnight admissions from both night float residents and nocturnists. Questions pertained to the quality and safety of information transmission and the educational experience of receiving providers. Surveys were designed based on prior literature, personal experience, and expert suggestion and refined in works in progress meetings (16-18). Survey responses were Likert-style format, querying respondents’ level of agreement (1-5, strongly disagree to strongly agree) with statements. Surveys were disseminated via e-mail by one of the study investigators (GD), with embedded informed consent and a link that connected anonymously to the online survey (Qualtrics, Provo, UT). We did not collect unique identifiers and offered no incentive for participation. Data were downloaded to a secure database and analyzed using JMP 14 software (SAS Institute, Inc., Cary, North Carolina). Data are presented as median ± interquartile range. Comparisons between medians were done by the Wilcoxon test. For all data, a *P-*value of ≤ 0.05 was considered statistically significant. The study was approved by the institutional review board.

## Results

A total of 108 providers responded (41% response rate) to the survey with five participants excluded for not having received overnight handoffs. Of the remaining 103 participants, there were 29 faculty and 74 residents. Attending physicians had an average age of 45 (±12.7) years, with a median experience of five years. Of faculty respondents, 13 were hospitalists, three were general internists, 10 were subspecialists, and three declined to respond. Resident physicians had an average age of 29.8 (±2) years and included 29 post-graduate year 1 (PGY1), 27 PGY2, 18 PGY3, and 2 PGY4.

Quality and safety of the handoff

When comparing housestaff and attendings responses, residents preferred receiving signout from the overnight resident, while attendings preferred signout from the nocturnist (Table 1). Attendings better understood “to dos” (specific uncompleted tasks) when receiving signout from nocturnists and reported that nocturnists better communicated pending tests and were more likely to complete medication reconciliation. Housestaff felt that the overnight residents were more likely to model professional behavior during the handoff, while attendings reported no difference in professional behavior between the groups.

**Table 1:** A comparison between resident and attending perceptions of (A) resident and (B) nocturnist handoffs with respect to quality & safety of transmission of information, responses scaled from 1-5, (strongly disagree to strongly agree), median (interquartile range)

A) Resident Handoff	Records Summarized	Overnight Events Communicated	“To Dos” Understood	Professional Behavior Modeled	Handoff Efficient	Prefer Handoff from Resident	Med Rec Complete	Pending Tests were Communicated
Residents	3.0 (3.0-4.0)	4.0 (4.0-4.0)	4.0 (4.0-4.0)	5.0 (4.0-5.0)	3.0 (3.0-4.0)	4.0 (3.0-4.0)	3.0 (3.0-4.0)	4.0 (3.0-4.0)
Attendings	4.0 (3.0-4.0)	4.0 (3.0-4.0)	4.0 (3.0-4.0)	4.0 (4.0-5.0)	3.0 (3.0-4.0)	3.0 (1.75-4.0)	3.5 (2.75-4.0)	4.0 (4.0-4.0)
p-value	0.97	0.49	0.2	0.03	0.78	<0.001	0.21	0.4
B) Nocturnist Handoff	Records Summarized	Overnight Events Communicated	“To Dos” Understood	Professional behavior modeled	Efficient	Prefer Nocturnist	Med Rec Complete	Pending Tests were Communicated
Residents	4.0 (3.0-4.0)	4.0 (4.0-4.0)	4.0 (4.0-4.0)	4.0 (4.0-5.0)	4.0 (4.0-4.0)	2.0 (2.0-3.0)	3.0 (3.0-4.0)	4.0 (3.0-4.0)
Attendings	4.0 (3.0-4.0)	4.0 (4.0-5.0)	4.0 (4.0-5.0)	4.0 (4.0-5.0)	4.0 (4.0-5.0)	4.0 (3.0-5.0)	4.0 (3.0-4.0)	4.0 (4.0-4.0)
p-value	0.07	0.07	0.003	0.85	0.18	<0.001	0.009	<0.001

Educational value of the handoff

Relative to receiving attendings, residents reported less time to ask questions and less comfort asking questions of the nocturnist during handoff (Table 2). Residents were less comfortable than attendings changing a nocturnist’s plan of care. Housestaff reported that receiving signout from the overnight resident was more likely to improve their confidence managing similar conditions. Attendings were more likely to feel ownership of the patient than residents were, irrespective of whether handoff came from a nocturnist or overnight resident. 

**Table 2:** Resident and attending perceptions of (A) resident and (B) nocturnist handoffs with respect to educational value of the handoff, responses scaled from 1-5, (strongly disagree to strongly agree), median (interquartile range)

A) Resident Handoff	I am Comfortable Asking Questions	I Have Time to Ask Questions	Handoff Improved my Confidence Managing Similar Conditions	I Feel Ownership of the Patient	I am Comfortable Changing the Plan	Care Demonstrated Evidence-based Medicine
Residents	5.0 (4.0-5.0)	4.0 (4.0-5.0)	4.0 (3.0-4.0)	4.0 (4.0-5.0)	5.0 (4.0-5.0)	4 (4-4)
Attendings	5.0 (4.75-5.0)	5.0 (3.0-5.0)	3.0 (2.0-3.0)	5.0 (3.75-5.0)	5.0 (4.0-5.0)	4 (4-4)
p-value	0.55	0.37	<0.001	0.03	0.64	0.75
B) Nocturnist Handoff	Comfortable asking questions	Time to ask questions	Improved my confidence	Ownership of the case	Comfortable changing the plan	Evidence-based
Residents	4.0 (3.0-4.75)	4.0 (3.0-4.0)	3.0 (3.0-4.0)	4.0 (3.0-5.0)	4.0 (3.0-5.0)	4 (4-4)
Attendings	5.0 (4.0-5.0)	5.0 (4.0-5.0)	3.0 (3.0-4.0)	5.0 (4.25-5.0)	5.0 (4.0-5.0)	4 (4-5)
p-value	<0.001	<0.001	0.55	<0.001	0.009	0.49

## Discussion

Despite positive faculty views of the nocturnist handoff and the potential benefits to trainees of faculty role-modeling, residents still preferred receiving signout from their overnight colleagues. Integration of practicing overnight faculty into the training environment may come at an educational cost, as housestaff felt they had less time to ask questions of the nocturnist, were less likely to change care plans, and that nocturnist signout was less likely to improve their confidence managing similar cases. The use of overnight attending physicians to perform direct patient care activities may contribute to scenarios where trainees feel unable to question management plans during the patient handoff, impairing reassessment and predisposing to anchor bias. Training programs should consider building systems that, when feasible, give preference to overnight residents presenting new admissions to teaching teams, as housestaff reported such handoffs improved their confidence managing similar conditions, provided adequate time for questions, and encouraged patient ownership.

In 2013, Dhaliwal and Hauer wrote on the need to update the format and content of the overnight admission presentation in the era of prevalent nigh float systems [19]. As we adapt the traditional oral patient presentation to current practice, our study emphasizes the importance of considering nocturnists in this restructuring. Given the demonstrated reluctance of trainees to ask questions of attendings or change care plans, when nocturnists do present new patients to teams of trainees, a deliberate structure should mandate dedicated time for housestaff questions and proactive trainee reevaluation of the working diagnosis.

In addition, our study highlights potential differences between overnight attending and resident presentations with regard to the quality of information transmission. To eliminate handoff disparities, programs should consider constructing scripts for morning presentations that include overnight “always events” (items such as medication reconciliation status, pending tests, and incomplete tasks). Standardization of both the form and content of morning handoffs may help mitigate handoff errors.

There are several limitations to our study. First, the findings represent the experience of internal medicine residents and attendings at a single academic, safety-net hospital and may not be generalizable to other institutions or specialties. Second, we asked respondents to recall patient handoffs from the prior year, which may have introduced recall bias. Third, responses of junior and senior housestaff were pooled, which may weight responses in favor of higher responding groups (more junior trainees, in our study). Finally, we reported only resident and attending perceptions of the quality, safety, and educational value of the overnight admission handoff, and not direct measures of knowledge or patient care outcomes. Further research should explore patient-level clinical outcomes for patients admitted by overnight residents and nocturnists as well as direct measures of difference in knowledge acquisition from overnight resident and attending handoffs.

## Conclusions

With increasingly prevalent night float systems and nocturnist providers, academic programs have to negotiate balancing safe and high-quality patient care with creating positive learning environments and clear expectations. A modern approach to handoffs of overnight admissions involves standardizing core content for presentations and ensuring a structure containing dedicated time for trainees’ questions and diagnostic reassessment.
